# Aseptic Surgery

**Published:** 1909-09-11

**Authors:** 


### Surgery.

### ASEPTIC SURGERY.

The last few years have witnessed a gradual but steady increase in the popularity of aseptic as opposed to antiseptic surgery. This has been a gradual evolution, and we can trace the various steps by which it has been brought about. Lord Lister in this country found that certain strong chemical compounds had the effect of destroying these micro-organisms, and devised methods by which he claimed suppuration could be reduced to a minimum. He met with the opposition which is the experience of all reformers; but eventually he gained the day, though it was some years before his teaching was universally accepted. This was the result of two factors: his original methods, *e.g.* the carbolic spray, etc., were admittedly inadequate; but further, imperfect as they were, they were not properly applied by the surgeons who first used them. One hears, for instance, stories of surgeons in the early days of antiseptic surgery who operated with a carbolic spray, but omitted to cleanse their hands, and then published their results to prove that Lord Lister's work was incorrect.

In spite of these early difficulties, however, antiseptic surgery made the progress it was bound to do, and a new variety of antiseptic was constantly discovered. Of these, carbolic acid and the salts of mercury, either mercury perchloride or mercuric potassium iodide were the ones that came into general use.

By their means a very high percentage of operations without suppuration could be guaranteed; but they had certain disadvantages. Cases of poisoning by mercury after irrigating large suppurating cavities with corrosive sublimate or biniodide were reported; and it was argued that the introduction of such powerful chemicals into the peritoneum, for instance, was likely to diminish its vitality. Some means was therefore sought by which pyogenic organisms could be eliminated from the field of operation without as far as possible the aid of chemical re-agents. Thus the rise of aseptic surgery.

Heat of a sufficiently high degree is admittedly the best of all means of sterilisation, and everything connected with a surgical operation can be submitted to this with the exception of the patient's skin, the surgeon's hands, and certain foıms of ligature. All instruments to be used must be boiled. Some surgeons say that to boil a knife destroys its edge. This is partially true, but one boiling is not sufficient to make it useless, and after each operation it should be reset. Those who object to boiling scalpels usually pass them through a carbolic solution 1 in 20 and keep them in methylated spirit, but this is a departure from the aseptic method.

All dressings, towels, swabs, etc., should be placed in specially made drums, with a visor arrangement which can be opened during the sterilising process and shut afterwards. The drums are placed in an autoclave and heated to 115° C., their contents being thus rendered absolutely sterile. The mackintoshes which are placed on the patient should be subjected to the same treatment. This can easily be done by employing instead of ordinary mackintoshes large sheets of specially prepared jaconet.

One great advantage of this system is that it is not absolutely essential to carry about large trays to place one's instruments in. A sheet of the sterilised jaconet may be laid over a table and on this again a sterilised towel on which all instruments can be put when they are taken out of the steriliser. If it is preferred to keep them in fluid, sterilised normal saline solution is the best to employ.

With regard to the surgeon's hands, much controversy has ranged round the question of india-rubber gloves. Arguments have been adduced against them that they impede the operator and increase the time of the operation; that if a glove is accidentally pricked by a needle there is a chance of the wound becoming thus contaminated. In the writer's experience it is not more difficult to operate with gloves when one is once accustomed to them, provided that the gloves fit well and are properly put on. The best way of putting them on is to fill them with saline solution, insert the hand, and then hold up the hand so as to allow the fluid to run out at the wrist. Some surgeons prefer to cover the hands with liquid soap and then put on the gloves. This is an easy method, but when the glove is on it does not adhere firmly to the fingers, and the difficulty of the operation is undoubtedly increased thereby. The argument that accidental pricking of the glove may lead to contamination of the wound is met by the rule that the hands must always be cleansed as carefully as if one was about to operate without gloves.

In one particular only are we now unable to dispense with chemical antiseptics, and that is in the preparation of the patient's skin; and to ensure successful asepsis this is particularly important. The success of operations where asepsis is absolutely essential to the welfare of the patient, such as in opening the knee-joint, depends largely on the thoroughness with which this is done. It is well, therefore, to begin the preparation two or three days before the operation by shaving the part if necessary and then thoroughly cleansing it with soap and water. It should next be rubbed with ether to get rid of fatty débris, and swabbed over with a solution of mercuric potassium iodide in spirit. Finally it should be wrapped in a biniodide or carbolic dressing covered with jaconet to keep it moist, which should be changed daily until the day of operation.

If all these precautions are observed the percentage of clean cases which suppurate can be reduced to a minimum. The individual factor varies with every surgeon, but in most tables of statistics of operations performed aseptically the number of cases which suppurate amount to less than 5 per cent.

